# Supplementary material for: The population genetics of the causative agent of snake fungal disease indicate recent introductions to the USA
Source: PLoS Biol. 2022 Jun 23;20(6):e3001676. doi: 10.1371/journal.pbio.3001676 (PMC9223401; doi:10.1371/journal.pbio.3001676)
Supplement: S1 Fig — (A) Maximum-likelihood phylogeny including all 82 Oo strains and based on an amino acid-level alignment consisting of 5,811 nuclear proteins and 3,311,400 positions. (B) Boxplot showing the mean, pairwise percent divergence between Clade III and Clades I and II across the 5,811 proteins included in the alignment for (A). The red dashed lines indicate the values for the 2 protein coding genes that have been used previously in phylogenetic studies of Oo: actin (ACT) and translation elongation factor 2ɑ (TEF). The limits of the box correspond to the first and third quartiles, the black line inside the box corresponds to the median, and the whiskers extend to points that lie within 1.5 interquartile ranges of the first and third quartiles. (C) Maximum-likelihood phylogeny including all 82 Oo strains and based on a reference-based, nucleotide-level mitochondrial alignment (50,624 positions). In both (A) and (C), tip shapes indicate whether the infected snake was in captivity (triangles) or wild (circles). Filled black and white circles indicate nodes with bootstrap support ≥90 and ≥70, respectively. Gray vertical lines and labels indicate the 3 primary clades. Data underlying this figure can be found in OSF: https://osf.io/fmbh5/. (PDF) [file pbio.3001676.s001.pdf]

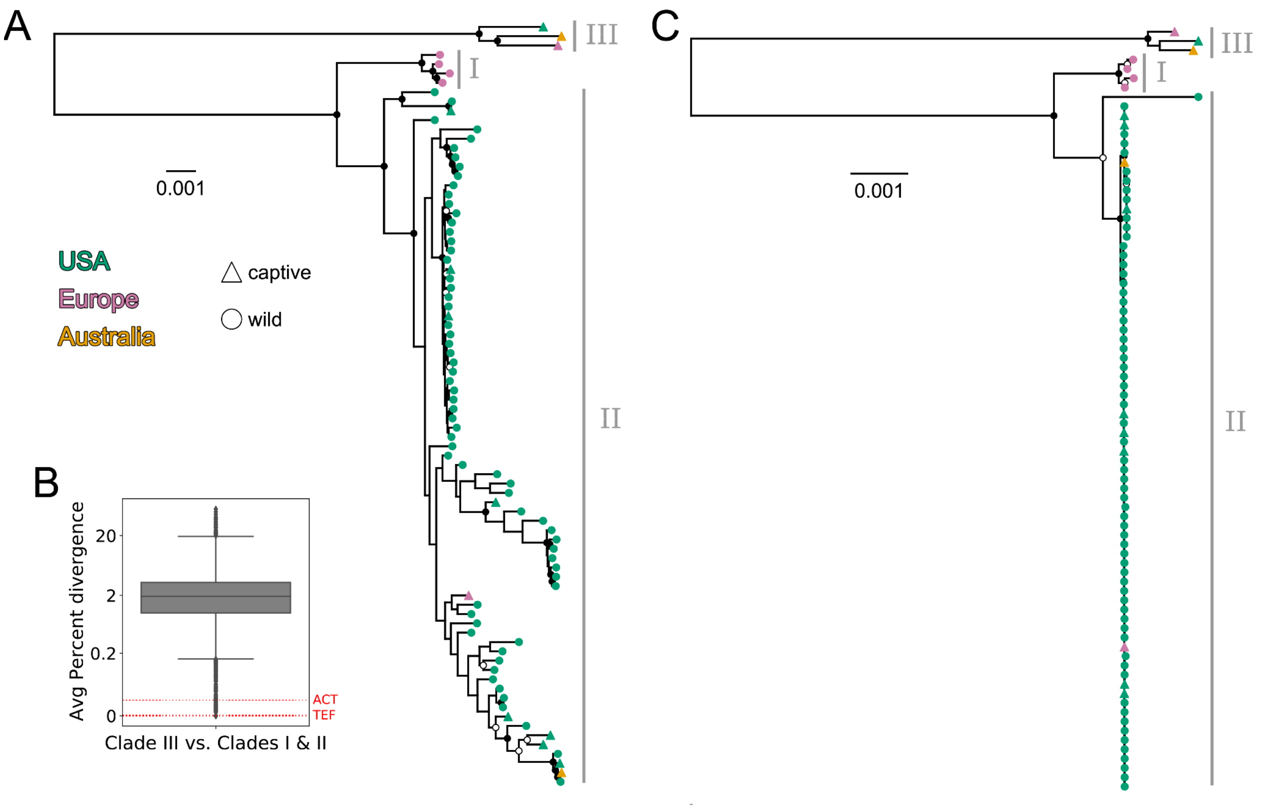

S1 Fig. All North American *Ophidiomyces ophidiicola* (*Oo*) strains isolated from wild snakes belong to a distinct phylogenetic clade. (A) Maximum-likelihood phylogeny including all 82 *Oo* strains and based on an amino acid-level alignment consisting of 5,811 nuclear proteins and 3,311,400 positions. (B) Boxplot showing the mean, pairwise percent divergence between Clade III and Clades I and II across the 5,811 proteins included in the alignment for (A). The red dashed lines indicate the values for the two protein coding genes that have been used previously in phylogenetic studies of *Oo*: actin (*ACT*) and translation elongation factor 2α (*TEF*). The limits of the box correspond to the 1st and 3rd quartiles, the black line inside the box corresponds to the median, and the whiskers extend to points that lie within 1.5 interquartile ranges of the 1st and 3rd quartiles. (C) Maximum-likelihood phylogeny including all 82 *Oo* strains and based on a reference-based, nucleotide-level mitochondrial alignment (50,624 positions). In both (A) and (C), tip shapes indicate whether the infected snake was in captivity (triangles) or wild (circles). Filled black and white circles indicate nodes with bootstrap support ≥90 and ≥70, respectively. Gray vertical lines and labels indicate the three primary clades. Data underlying this figure can be found in OSF: https://osf.io/fmbh5/.
