# Supplementary material for: The population genetics of the causative agent of snake fungal disease indicate recent introductions to the USA
Source: PLoS Biol. 2022 Jun 23;20(6):e3001676. doi: 10.1371/journal.pbio.3001676 (PMC9223401; doi:10.1371/journal.pbio.3001676)
Supplement: S2 Fig — Log-transformed Phi test p-values calculated using PhiPack with a window size of 50,000 bases and a step size of 25,000 bases (1). Black horizontal dashed line indicates a p-value of 0.05 with Bonferroni correction for 749 different tests. Red vertical dashed lines indicate scaffold boundaries within the reference assembly. Data underlying this figure can be found in OSF: https://osf.io/fmbh5/. (PDF) [file pbio.3001676.s002.pdf]

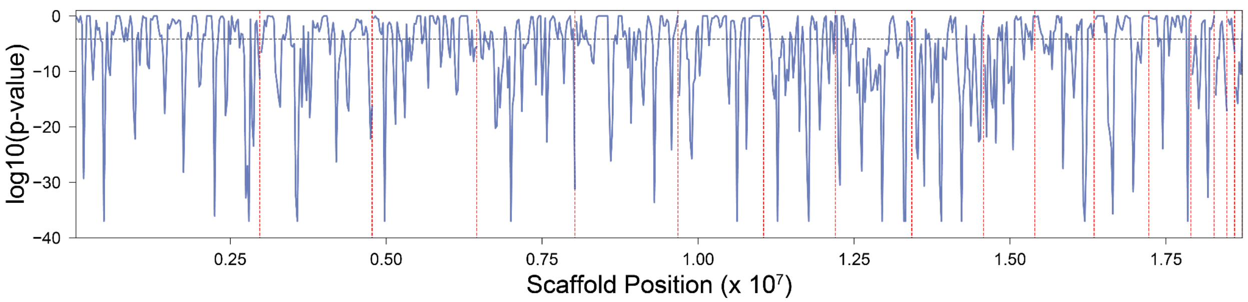

S2 Fig. Evidence for recombination within Clade II throughout the *Ophidiomyces ophidiicola* genome. Log-transformed Phi test p-values calculated using PhiPack with a window size of 50,000 bases and a step size of 25,000 bases (1). Black horizontal dashed line indicates a p-value of 0.05 with Bonferroni correction for 749 different tests. Red vertical dashed lines indicate scaffold boundaries within the reference assembly. Data underlying this figure can be found in OSF: https://osf.io/fmbh5/.
