# Supplementary material for: The population genetics of the causative agent of snake fungal disease indicate recent introductions to the USA
Source: PLoS Biol. 2022 Jun 23;20(6):e3001676. doi: 10.1371/journal.pbio.3001676 (PMC9223401; doi:10.1371/journal.pbio.3001676)
Supplement: S3 Fig — Solid circles represent nonrecombinant clonal lineages. Divided circles depict recombinant strains (color combinations qualitatively indicate genetic signatures of the various lineages present in those recombinants [see Fig 2]). For strain NWHC 44736–75, a portion of the circle is gray to indicate an unsampled parent lineage. Circle sizes denote the number of strains when multiple strains were collected in proximity to one another. The edge of the Atlantic and Gulf Coastal Plain region is shown with a dashed line. Data underlying this figure can be found in OSF: https://osf.io/fmbh5/. The base map used to generate this figure is from Natural Earth (https://www.naturalearthdata.com) and available through GitHub (https://github.com/nvkelso/natural-earth-vector/blob/master/geojson/ne_50m_admin_1_states_provinces_lakes.geojson). (PDF) [file pbio.3001676.s003.pdf]

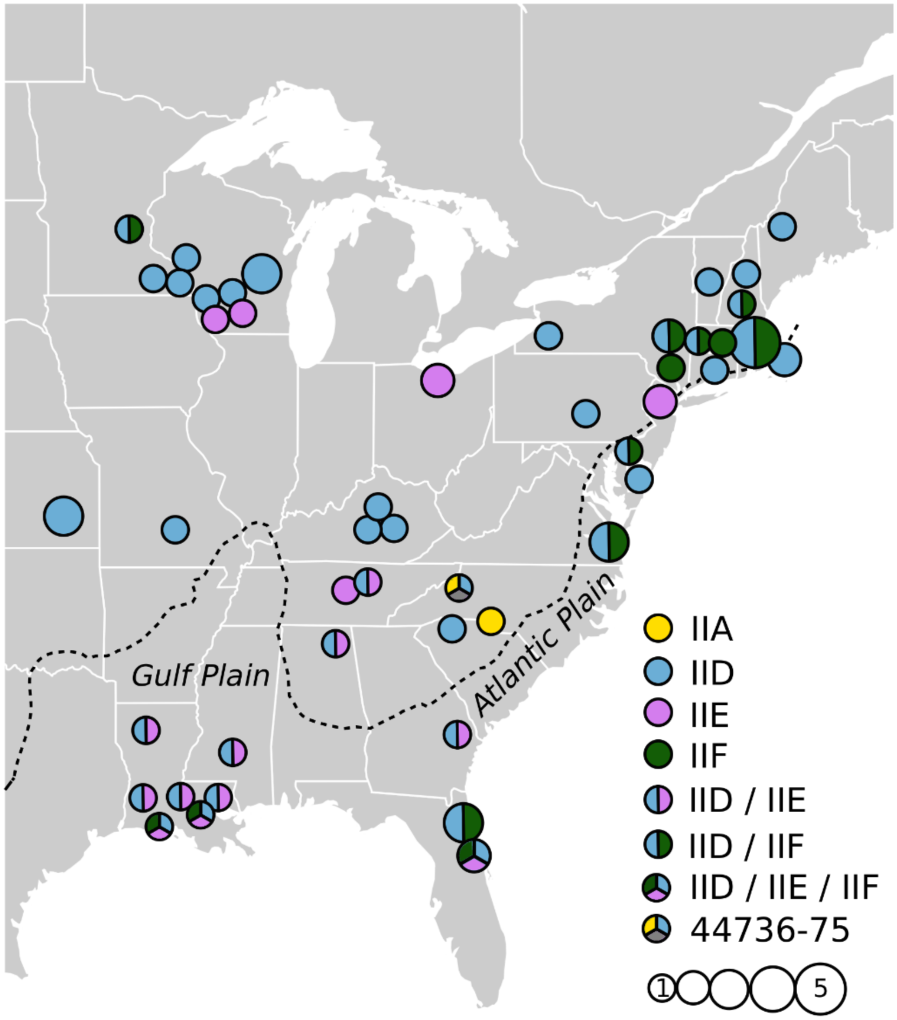

S3 Fig. Geographic distribution of *Ophidiomyces ophidiicola* strains isolated from wild snakes in the eastern USA. Solid circles represent non-recombinant clonal lineages. Divided circles depict recombinant strains (color combinations qualitatively indicate genetic signatures of the various lineages present in those recombinants [see Fig. 2]). For strain NWHC 44736-75, a portion of the circle is gray to indicate an unsampled parent lineage. Circle sizes denote the number of strains when multiple strains were collected in proximity to one another. The edge of the Atlantic and Gulf Coastal Plain region is shown with a dashed line. Data underlying this figure can be found in OSF: https://osf.io/fmbh5/. The base map used to generate this figure is from Natural Earth (https://www.naturalearthdata.com) and available through GitHub (https://github.com/nvkelso/natural-earth-vector/blob/master/geojson/ne_50m_admin_1_states_provinces_lakes.geojson).
