# Supplementary material for: The population genetics of the causative agent of snake fungal disease indicate recent introductions to the USA
Source: PLoS Biol. 2022 Jun 23;20(6):e3001676. doi: 10.1371/journal.pbio.3001676 (PMC9223401; doi:10.1371/journal.pbio.3001676)
Supplement: S4 Fig — Great-circle distance (x-axis) versus pairwise nucleotide divergence (y-axis) for each pair of strains that were isolated from wild snakes and belonged to the same clonal lineage within Clade II. Each point represents a pair of strains, and the colors indicate the clonal lineages. Genetic divergence was calculated using 116,322 positions in the nuclear genome that were variable among Oo strains from Clade II. Data underlying this figure can be found in OSF: https://osf.io/fmbh5/. (PDF) [file pbio.3001676.s004.pdf]

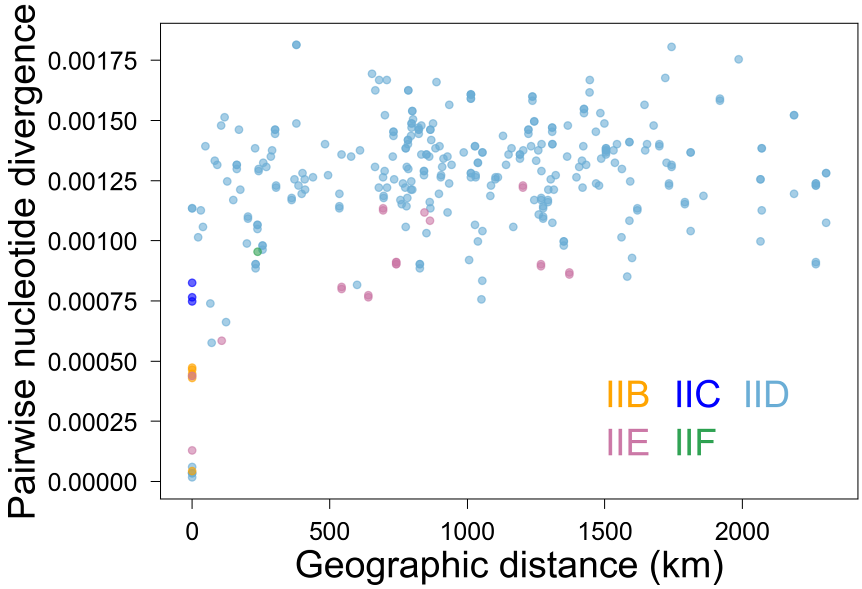

S4 Fig. Within clonal lineages of *Ophidiomyces ophidiicola* (*Oo*), there is no evidence of recent, long-distance dispersal. Great-circle distance (x-axis) versus pairwise nucleotide divergence (y-axis) for each pair of strains that were isolated from wild snakes and belonged to the same clonal lineage within Clade II. Each point represents a pair of strains and the colors indicate the clonal lineages. Genetic divergence was calculated using 116,322 positions in the nuclear genome that were variable among *Oo* strains from Clade II. Data underlying this figure can be found in OSF: https://osf.io/fmbh5/.
