# Supplementary material for: The population genetics of the causative agent of snake fungal disease indicate recent introductions to the USA
Source: PLoS Biol. 2022 Jun 23;20(6):e3001676. doi: 10.1371/journal.pbio.3001676 (PMC9223401; doi:10.1371/journal.pbio.3001676)
Supplement: S5 Fig — Root-to-tip divergence (y-axis) was calculated using TempEst (2) v1.5.3 using maximum-likelihood phylogenies, and the colored numbers inside each plot represent Pearson correlation coefficients. The mitochondrial tree (left) was rooted using the residual mean squared best-fitting root function. The nuclear trees for the Clade II clonal lineages (right) were rooted using the correlation best-fitting root function. A positive slope indicates the presence of a molecular clock signal. Data underlying this figure can be found in OSF: https://osf.io/fmbh5/. (PDF) [file pbio.3001676.s005.pdf]

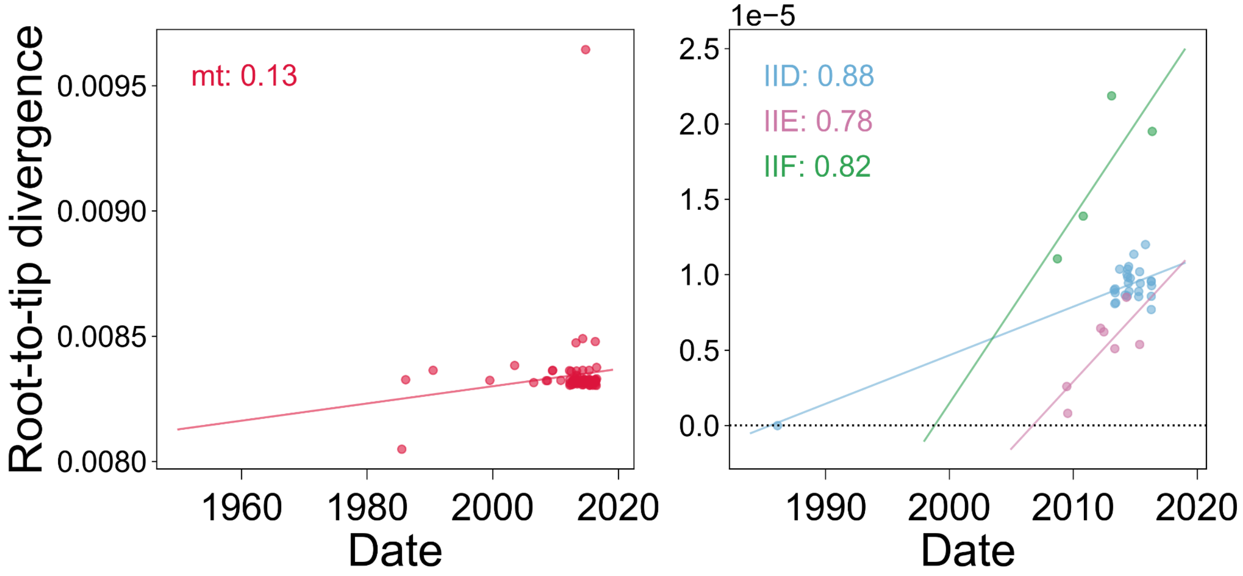

S5 Fig. Root-to-tip plots demonstrate the presence of molecular clock signals within both the mitochondrial and nuclear genomes of *Ophidiomyces ophidiicola*. Root-to-tip divergence (y-axis) was calculated using TempEst (2) v1.5.3 using maximum-likelihood phylogenies and the colored numbers inside each plot represent Pearson correlation coefficients. The mitochondrial tree (left) was rooted using the residual mean squared best-fitting root function. The nuclear trees for the Clade II clonal lineages (right) were rooted using the correlation best-fitting root function. A positive slope indicates the presence of a molecular clock signal. Data underlying this figure can be found in OSF: https://osf.io/fmbh5/.
