# Supplementary material for: The population genetics of the causative agent of snake fungal disease indicate recent introductions to the USA
Source: PLoS Biol. 2022 Jun 23;20(6):e3001676. doi: 10.1371/journal.pbio.3001676 (PMC9223401; doi:10.1371/journal.pbio.3001676)
Supplement: S6 Fig — Colored distributions represent the posterior probability distributions from BEAST for the Oo substitution rate in number of substitutions per site per year. There is a single rate estimate for the mitochondrial genome based on a strict clock model (top, red) and 6 rate estimates for the nuclear genome, 2 for each of the 3 primary clonal lineages based on the strict (solid line) and relaxed (dotted line) clock models. Hatched regions represent the tails of each distribution that fall outside of the 95% highest posterior density. Gray boxes indicate published rate estimates for Batrachochytrium dendrobatidis (Bd) (3), with the edges of the boxes indicating the boundaries of the 95% highest posterior densities and the dashed lines representing the mean estimates. Data underlying this figure can be found in OSF: https://osf.io/fmbh5/. (PDF) [file pbio.3001676.s006.pdf]

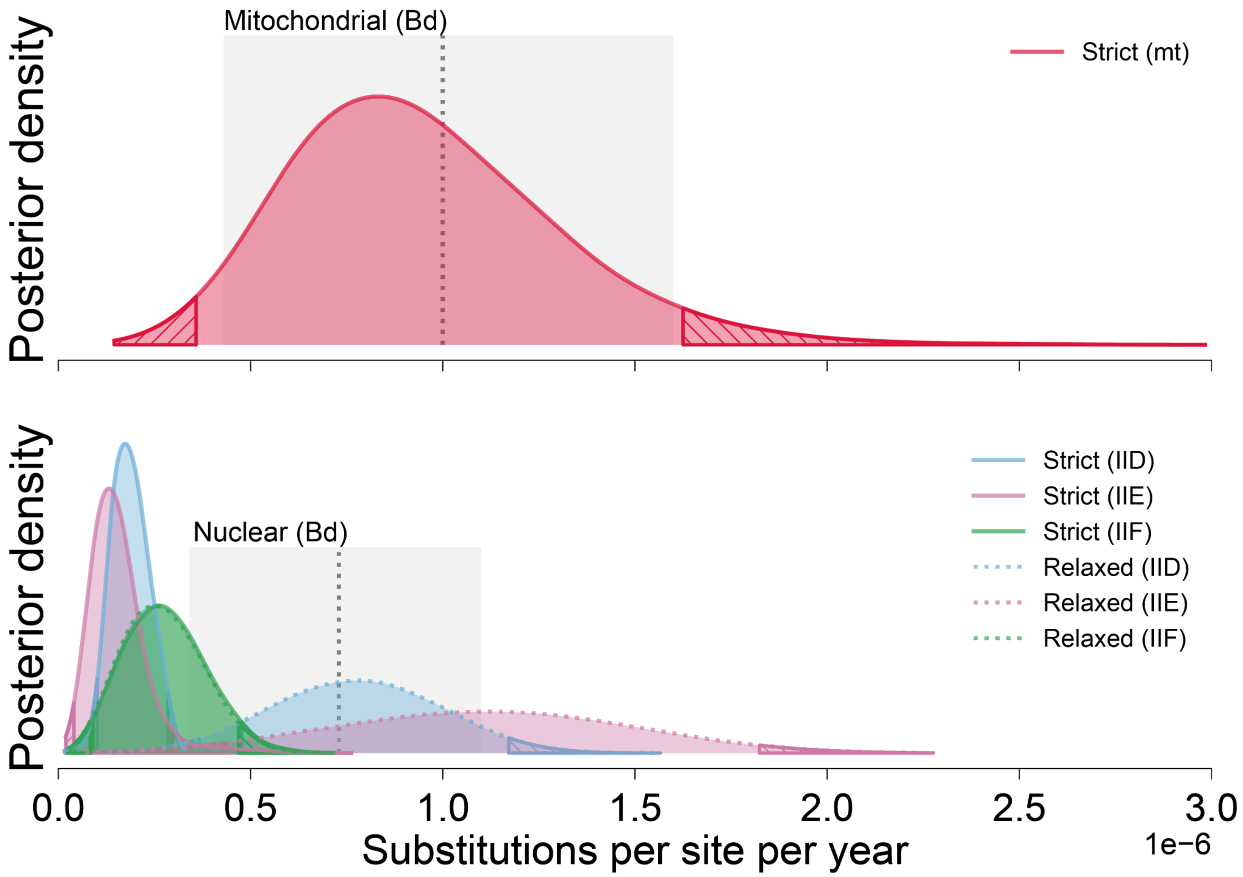

S6 Fig. Substitution rate estimates for *Ophidiomyces ophiodiicola* (*Oo*) are very similar to published estimates for *Batrachochytrium dendrobatidis* (*Bd*). Colored distributions represent the posterior probability distributions from BEAST for the *Oo* substitution rate in number of substitutions per site per year. There is a single rate estimate for the mitochondrial genome based on a strict clock model (top, red) and six rate estimates for the nuclear genome, two for each of the three primary clonal lineages based on the strict (solid line) and relaxed (dotted line) clock models. Hatched regions represent the tails of each distribution that fall outside of the 95% highest posterior density. Gray boxes indicate published rate estimates for *Batrachochytrium dendrobatidis* (*Bd*) (3), with the edges of the boxes indicating the boundaries of the 95% highest posterior densities and the dashed lines representing the mean estimates. Data underlying this figure can be found in OSF: https://osf.io/fmbh5/.
