# Supplementary material for: The population genetics of the causative agent of snake fungal disease indicate recent introductions to the USA
Source: PLoS Biol. 2022 Jun 23;20(6):e3001676. doi: 10.1371/journal.pbio.3001676 (PMC9223401; doi:10.1371/journal.pbio.3001676)
Supplement: S7 Fig — Both Bayesian and maximum-likelihood analyses produced trees with identical topologies (consensus tree from Bayesian analysis is shown). Posterior probabilities/bootstrap support values are shown at each node. Note that 2 strains recovered from wild snakes in Taiwan (shown in yellow) are most closely related to Clade III and 2 strains reside within Clade II (i.e., North American clade). Data underlying this figure can be found in OSF: https://osf.io/fmbh5/. (PDF) [file pbio.3001676.s007.pdf]

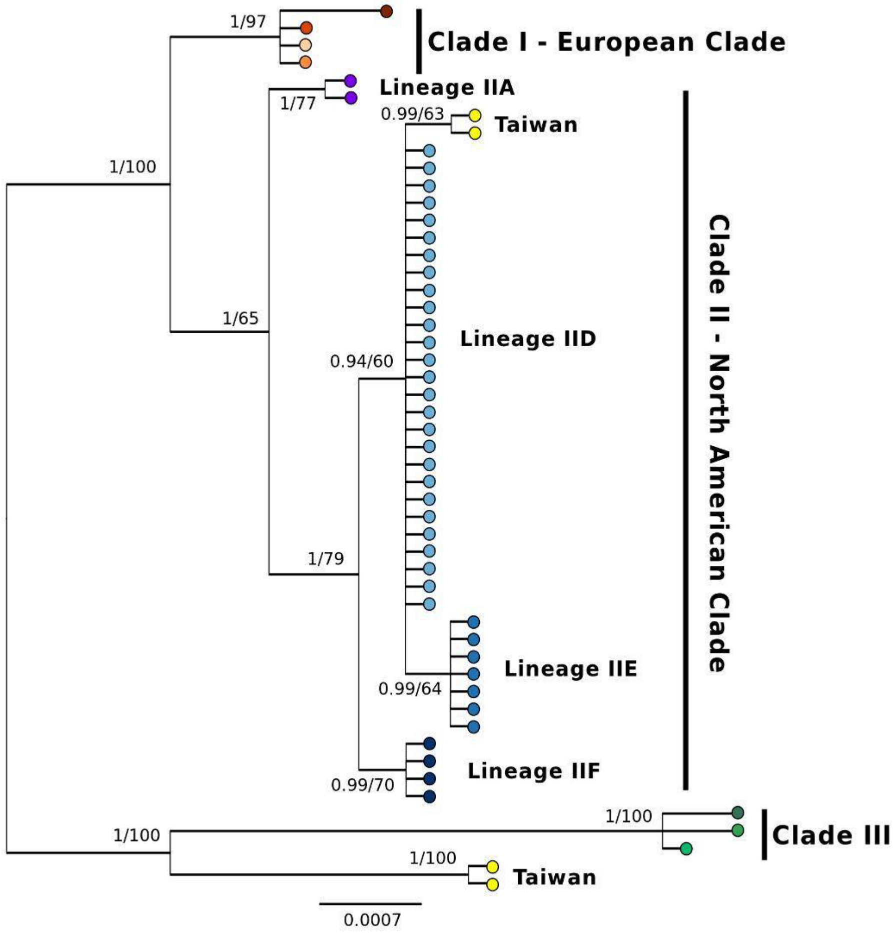

S7 Fig. Phylogenetic tree of *Ophidiomyces ophidiicola* strains, including all strains from Clades I and III, nonrecombinant strains from Clade II, and strains isolated from wild snakes in Taiwan, based on three concatenated loci (internal transcribed spacer region, actin, and translation elongation factor 2α). Both Bayesian and maximum likelihood analyses produced trees with identical topologies (consensus tree from Bayesian analysis is shown). Posterior probabilities/bootstrap support values are shown at each node. Note that two strains recovered from wild snakes in Taiwan (shown in yellow) are most closely related to Clade III and two strains reside within Clade II (i.e., North American clade). Data underlying this figure can be found in OSF: https://osf.io/fmbh5/.
