# Supplementary material for: The population genetics of the causative agent of snake fungal disease indicate recent introductions to the USA
Source: PLoS Biol. 2022 Jun 23;20(6):e3001676. doi: 10.1371/journal.pbio.3001676 (PMC9223401; doi:10.1371/journal.pbio.3001676)
Supplement: S1 File — (PDF) [file pbio.3001676.s013.pdf]

S1 File. Supporting results and methods

Mating type locus PCR assays

Results

All nine European isolates (NWHC 45692-12, NWHC 45707-84, NWHC 46078-5, NWHC 46078-6, NWHC 46078-7, NWHC 46078-8, NWHC 46078-9, NWHC 46078-10, NWHC 46078-11) screened with the primers were positive for only the *MAT1-2* locus. Amplicons were sequenced in both directions and were identical to the sequences of *MAT1-2* generated through whole genome sequencing of the other European isolates.

Methods

The mating type loci present in nine European isolates of *Ophidiomyces ophidiicola* (*Oo*) that were not included in the whole genome sequencing analysis were determined using newly designed PCR assays. Sequences of the *MAT1-1* and *MAT1-2* loci from whole genome sequence data of *Oo* were aligned to identify conserved regions. The program Primer3 (4-5) was then used to design two pairs of primers. The first primer pair (Oo MAT1-1 F: 5' - GAAAGTTAAATCGGGCTTG - 3'; Oo MAT1-1 R: 5' - TGGATGAATAGCGGTAGG - 3') targeted a 574 nucleotide portion of the *MAT1-1* locus. The second primer pair (Oo MAT1-2 F: 5' - CAATAGGTTGGTGCTGGT - 3'; Oo MAT1-2 R: 5' - GTCCCAGTCGTTGCTTTC - 3') targeted a 1,013 nucleotide portion of the *MAT1-2* locus.

The primer pairs were validated by screening 12 strains of *Oo* for which mating type was previously determined (five strains containing *MAT1-1*, seven strains containing *MAT1-2*) based on whole genome sequence data. Each reaction consisted of 23.75 µL water, 10 µL GoTaq Flexi

Buffer (Promega Corporation, Madison, Wisconsin), 5 µL 2.5 mM each dNTPs, 3 µL 25 mM $MgCl_2$, 2.5 µL 20 µM forward primer, 2.5 µL 20 µM reverse primer, 0.25 µL GoTaq DNA polymerase, and 3 µL extracted DNA template. Cycling conditions for the PCR were as follows: 95°C for 5 min; 45 cycles of 94°C for 45 sec, 52°C for 45 sec, 72°C for 1 min; 72°C for 10 min. All strains predetermined to contain the *MAT1-1* or *MAT1-2* locus yielded an amplicon with the correct primer set and of the expected size.

Phylogenetic analysis including Asian isolates of *Ophidiomyces ophidiicola*

Results

The four Asian isolates originated from two snakes (two isolates per individual snake). In both cases, the two isolates obtained from the same snake had identical sequences for the three loci examined. However, two unique genotypes were identified. Consensus trees resulting from the Bayesian and maximum likelihood analyses had identical topologies and good support for most of the clades and clonal lineages. One of these Asian strains (strain 2605) possessed 13 unique single nucleotide polymorphisms (SNPs) compared to the strains we sequenced. This genotype grouped with, yet was distinct from, Clade III from our analysis. The second Asian strain (strain 2664) resided within Clade II. This strain grouped with lineage IID but had three unique SNPs separating it from other isolates within IID (Fig. S7).

Methods

Oo was recently isolated from two wild snakes in Taiwan (6). However, DNA sequence data were only available for three loci (internal transcribed spacer region [ITS], actin [*ACT*], and translation elongation factor 2α [*TEF*]) from those isolates; thus, those isolates could not be

included in analyses using whole genome sequence data. To determine how the Asian isolates of *Oo* compared to the strains we examined, we performed a separate phylogenetic analysis by concatenating ITS, *ACT*, and *TEF* sequence data. In addition to the Asian isolates, we included all of our strains from Clades I and III and non-recombinant strains from Clade II. Sequence data for *ACT* and *TEF* were obtained from the *de novo* genome assemblies of each strain. The ITS is part of the rRNA gene complex which occurs as tandem repeats on the fungal genome and is often excluded from genome assemblies. Thus, we used ITS sequence data that was already in GenBank for a given strain or new sequence data was generated as described in previously (7). Newly generated sequences for the ITS were deposited in GenBank (see Table S2).

Sequence data for each locus were aligned using MUSCLE (8). All gaps were deleted from the alignments and data for the three loci were concatenated. For phylogenetic analyses, the loci were partitioned with a Kimura two-parameter model applied to the ITS and Hasegawa-Kishino-Yano (HKY) model applied to *ACT* and *TEF* data. A maximum likelihood analysis was performed in PAUP* v4.0a169 with 1,000 bootstrap iterations using a heuristic search technique and the sub-tree pruning and regrafting rearrangement operation. A Bayesian analysis was performed on the same dataset using MrBayes v3.2.7a through the CIPRES Science Gateway (9). For the Bayesian analysis, we conducted two runs with four chains and 5,000,000 generations, with a sampling frequency of 1,000 generations.

DISCLAIMER

Any use of trade, firm, or product names is for descriptive purposes only and does not imply endorsement by the U.S. Government.

SUPPORTING REFERENCES

1. Bruen TC, Philippe H, Bryant D. A simple and robust statistical test for detecting the presence of recombination. Genetics. 2006;172: 2665–2681. doi:10.1534/genetics.105.048975

2. Rambaut A, Lam TT, Max Carvalho L, Pybus OG. Exploring the temporal structure of heterochronous sequences using TempEst (formerly Path-O-Gen). Virus Evol. 2016;2: vew007.

3. O'Hanlon SJ, Rieux A, Farrer RA, Rosa GM, Waldman B, Bataille A, et al. Recent Asian origin of chytrid fungi causing global amphibian declines. Science. 2018;360: 621–627.

4. Koressaar T, Remm M. Enhancements and modifications or primer design program Primer 3. Bioinformatics. 2007;23: 1289-1291.

5. Untergasser A, Cutcutache I, Koressaar T, Ye J, Faircloth BC, Remm M, Rozen SG. Primer3 - new capabilities and interfaces. Nucleic Acids Res 2012;40: e115.

6. Sun P-L, Yang C-K, Li W-T, Lai W-Y, Fan Y-C, Huang H-C, et al. Infection with Nannizziopsis guarroi and Ophidiomyces ophiodiicola in reptiles in Taiwan. Transbound

Emerg Dis. 2022;69:764–775. doi:10.1111/tbed.14049

7. Lorch JM, Knowles S, Lankton JS, Michell K, Edwards JL, Kapfer JM, et al. Snake fungal disease: an emerging threat to wild snakes. Philos Trans R Soc Lond B Biol Sci. 2016;371: 20150457. doi:10.1098/rstb.2015.0457

8. Edgar RC. MUSCLE: multiple sequence alignment with high accuracy and high throughput. Nucleic Acids Res. 2004;32: 1792-1797.

9. Miller MA, Pfeiffer W, Schwartz T. Creating the CIPRES Science Gateway for inference of large phylogenetic trees *in* Proceedings of the Gateway Computing Environments Workshop (GCE), 14 Nov. 2010, New Orleans, LA pp 1-8.
