# Supplementary material for: The population genetics of the causative agent of snake fungal disease indicate recent introductions to the USA
Source: PLoS Biol. 2022 Jun 23;20(6):e3001676. doi: 10.1371/journal.pbio.3001676 (PMC9223401; doi:10.1371/journal.pbio.3001676)
Supplement: Attachment — Submitted filename: ResponseToReviews.docx [file pbio.3001676.s014.docx]

**We are grateful for the positive comments and feedback from the reviewers and editors. We believe these comments have helped to clarify and strengthen certain aspects of the manuscript and make it an overall better paper. Our responses to individual comments are below (our responses are in blue bold font). Please note that in addition to the suggestions by the reviewers and editor, we also made some minor grammatical changes and word use changes to this version of the manuscript based upon review of an official within our agency.**

Reviewer #1: In the paper "Population genetic analysis of Ophidiomyces ophidiicola, the causative agent of snake fungal disease, suggests recent introductions to the USA", the authors sequence 82 strains of the snake fungal disease Oo and perform genomic, population genetic and dating analysis to predict the timing and location of its emergence in the United States.

Overall, it is very nice study, with clear results regarding the population of Oo in north America. Correspondingly, I only have very minor suggestions for improvement.

**We are glad to hear that the reviewer shares our excitement for this study, and we thank them for their thoughtful comments.**

Firstly, given the authors have generated a new reference strain, I'd like to see a BUSCO gene completeness report for that (E.g. what percent of single copy orthologs were found?). It would also be nice to give further details about any previous genome assemblies that were available, and why these were deemed unsuitable / why they generated a new one?

**As suggested by the reviewer, we have added BUSCO gene completeness statistics to Table S1 (% complete, single, duplicate, fragmented and missing), along with several additional metrics useful for evaluating the completeness of an assembly (e.g., N50, L50, minimum and maximum contig lengths). Due to the increase in the number of columns, we are now providing this table as a separate file. We also added a sentence to the main text summarizing the BUSCO completeness statistics for our assemblies (Lines 143-147):**

**“A Benchmarking Universal Single-Copy Orthologs (BUSCO) analysis with 4,862 genes estimated that these assemblies were 91.1 - 93.4% complete (median = 92.9%), which is on par with the only *Oo* genome assembly currently present in NCBI (GenBank: MWKM00000000.1, 91.9% complete according to BUSCO)** [**[34]**](https://paperpile.com/c/pWh9e3/NWSpe)**.”**

**However, we would also like to clarify that our de novo nuclear assemblies and annotations were only used to generate the gene-level amino acid sequences that we used to generate a nuclear genome phylogeny including all 82 *Oo* isolates (Figures 1A and S1A in the revised version of our manuscript).**

**For our nuclear genome SNP analysis, we used the existing, publicly available *Oo* genome assembly, which consisted of 38 scaffolds (GenBank: MWKM01000001.1 - MWKM01000021.1, MWKM01000023.1 - MWKM01000039.1). The BUSCO completeness estimate for this assembly is on par with our own assemblies.**

**However, we did utilize a custom reference for our mitochondrial genome SNP analysis (GenBank: CM040671.1) because the mitochondrial scaffold from the existing, publicly available *Oo* genome assembly (GenBank: MWKM01000023.1) was incomplete, with 15 large gaps in contiguous sequence. Within the revised version of our manuscript, we have clarified this and explained our rationale for using a custom mitochondrial reference (Lines 599-604):**

**“For calling SNPs within the mitochondrial genome, we considered all 82 *Oo* strains and used a custom *de novo* assembly for the type strain of *Oo*, CBS 122913 (GenBank: CM040671.1), as a reference. We chose to use a custom reference for the mitochondrial genome because the mitochondrial scaffold for MYCO-ARIZ AN0400001 (GenBank: MWKM01000023.1) was incomplete, containing 15 large gaps in contiguous sequence.”**

Secondly, for variant calling with GATK, I'd like a proper description of the pipeline, including all commands used, and in particular, a description of any hard or soft filters used. The current description of variant calling would not be sufficient to replicate this study.

**As suggested by the reviewer, we have added a more detailed description of our variant calling pipeline to the methods section. The relevant paragraph can be found on Lines 590-612 of the revised manuscript and is copied here for convenience:**

**“We identified SNPs using NASP v1.2.0** [**[68]**](https://paperpile.com/c/pWh9e3/rI1TB) **with either Bowtie2 v2.4.2** [**[69]**](https://paperpile.com/c/pWh9e3/eKk0) **(mitochondrial genome) or BWA-mem v0.7.7** [**[70]**](https://paperpile.com/c/pWh9e3/hLNT) **(nuclear genome) to align reads (default settings) and with GATK v3.4-46-gbc02625** [**[71,72]**](https://paperpile.com/c/pWh9e3/RtyE+3FOB) **to call variants (-T UnifiedGenotyper -dt NONE -glm BOTH -out_mode EMIT_ALL_CONFIDENT_SITES -baq RECALCULATE -stand_call_conf 100 -ploidy 1). MUMmer v3.23** [**[73]**](https://paperpile.com/c/pWh9e3/hrA1) **was used to identify duplicated regions within our reference sequences (nucmer, find_duplicates). To avoid potentially erroneous genotype calls, we ignored any SNPs present within duplicated regions. For calling SNPs within the nuclear genome, we considered the 79 *Oo* strains from Clades I and II and utilized 38 scaffolds previously assembled from the MYCO-ARIZ AN0400001 strain of *Oo* as a reference (GenBank: MWKM01000001.1 - MWKM01000021.1 , MWKM01000023.1 - MWKM01000039.1).** **For calling SNPs within the mitochondrial genome, we considered all 82 *Oo* strains and used a custom *de novo* assembly for the type strain of *Oo*, CBS 122913 (GenBank: CM040671.1), as a reference. We chose to use a custom reference for the mitochondrial genome because the mitochondrial scaffold for MYCO-ARIZ AN0400001 (GenBank: MWKM01000023.1) is incomplete, containing 15 large gaps in contiguous sequence. For both the nuclear and mitochondrial genomes, we utilized all reference positions at which a high-confidence call was made for ≥80% of the included strains. Prior to running NASP, we removed sequencing adapters and trimmed low quality bases using BBDuk (last modified 2019-01-23) (bbduk.sh ktrim=r tpe tbo k=23 mink=8 hdist=1 hdist2=1 ftm=5 ref=~/bbmap/resources/adapters.fa; bbduk.sh qtrim=rl trimq=15 maq=20 ref=~/bbmap/resources/phix174_ill.ref.fa.gz k=31 hdist=1 minlen=50)** [**[53]**](https://paperpile.com/c/pWh9e3/JBDr0)**. We then generated maximum-likelihood phylogenies using RAxML-NG v0.5.1b** [**[74]**](https://paperpile.com/c/pWh9e3/PEzUM) **with the GTR+G model, 10 randomized parsimony starting trees, and 100 bootstrap replicates. For the mitochondrial SNP alignment, we also constructed a median-joining haplotype network** [**[75]**](https://paperpile.com/c/pWh9e3/SISDs) **using PopART** [**[76]**](https://paperpile.com/c/pWh9e3/zOQzB)**.”**

Finally, I think the tree with all clades i.e. clade III (currently in supplemental) should probably be added to Figure 1 (perhaps as part A, while the other 2 parts become B and C). I'm not certain what the rationale of having it supplemental is, other than it appearing much more distant.

**Our initial rationale for only including the tree with Clade III in the supplemental material is that the full version of that amino acid-based tree is somewhat redundant to the nucleotide-based tree shown in Figure 1. We also feel that the nucleotide-based tree provides a better resolution view of relationships within and among the strains of Clades I and II.**

**However, we agree with the reviewer that it is important to show the relationship of Clade III to Clades I/II in the main manuscript. Therefore, we have now included a simplified version of the Fig. S1 amino acid-based tree as a new panel in Figure 1, as suggested by the reviewer. In this version, the branches within Clade II have been collapsed to avoid redundancy with the nucleotide-level tree and to focus the amino acid-level tree on the relationships among the major Clades. The uncollapsed version of this tree is still available as a panel in Figure S1.**

For the Figure 1 legend, you should also describe what the branch lengths show / the length key.

**We have added a description of the branch length key to the Figure 1 legend (Lines 918-920): “In both (A) and (C), filled black circles indicate nodes with bootstrap support ≥90 and the scale bar represents the horizontal length equivalent to 0.001 changes per site.”**

Otherwise, this was an enjoyable and informative report on their work.

Reviewer #2: This is an important study on the population genetic history of an emerging fungal pathogen affecting a large and diverse group of wildlife. I think the work warrants close consideration by PLOS Biology.

**We thank the reviewer for their kind words and thoughtful comments.**

I really only have one significant concern, which is that the distinction between the two hypotheses seems somehow artificial. We understand the range of historical possibilities without arbitrarily designating these as two different hypotheses, although I recognize scientific writers differ in style sometimes to achieve the appearance of adherence to the relatively simplistic concepts of the "hypothetico-deductive method". The point is, I would welcome the authors to describe the historical possibilities with greater nuance and richness rather than adopt the binary either/or of the "novel pathogen hypothesis" vs "endemic pathogen hypothesis", especially since their own findings support a much more complicated pattern of introduction. Also, might it not be the case that the relatively recent detections of Oo are due to environmental change or stress, in which case the "emergence" of this disease should be causally attributed to both recent (post-colonial) introductions AND environmental changes?

**We completely agree that these binary categories are overly simplistic. In the manuscript we lay out these ‘competing hypotheses’ because they are widely applied to wildlife disease and are often important in assessing the threat level that a disease poses as well as the type of management approaches that are likely to be most effective for conservation purposes. In the introduction, we have rewritten the second paragraph to clarify this (Lines 69-80):**

**“Mechanisms for disease emergence are complex, but typically fall somewhere along a gradient between two broad categories: (1) introduction of an exotic pathogen into a naïve host population (“novel pathogen hypothesis”) or (2) *in situ* emergence of a native pathogen due to changes in environmental, host, or pathogen characteristics that alter disease ecology (“endemic pathogen hypothesis”) [10]. Although strict adherence to these binary categories may be an oversimplification in some cases (disease emergence mechanisms are often multifactorial), understanding whether a pathogen is native or introduced to a region is a key first step for assessing the threat of a disease to host populations and predicting pathogen spread, as well as for devising disease management strategies. For example, while targeted containment or eradication efforts may be effective against novel pathogens, successful management of endemic pathogens is more likely to involve manipulation of factors (e.g., environmental, host) that contribute to disease outbreaks [10].”**

**We refrain from further discussing the complexities of disease emergence in the introduction because our study did not examine other factors that might explain the emergence of SFD (i.e., we are simply asking the question of whether *Oo* is likely native to eastern North America or not). However, in the discussion section we have added the following sentences which explain that the novel and endemic pathogen hypotheses are not necessarily mutually exclusive (Lines 489-496):**

**“Recent changes in environmental conditions had previously been proposed to explain the potential emergence of SFD under the endemic pathogen hypothesis** [**[18]**](https://paperpile.com/c/pWh9e3/XRRAC)**, and these environmental changes may still be influencing prevalence or virulence, despite the relatively recent introduction of *Oo* to the USA. In other words, treating the novel and endemic pathogen hypotheses as competing explanations for SFD emergence may be overly simplistic. It is feasible that *Oo* could have been introduced into the eastern USA decades ago and emerged as an influential pathogen only recently due to changing environmental conditions and host health that caused a shift in host-pathogen ecology.”**

I also have a few questions that the study does not address, but which might be considered in the discussion.

- Why is Clade II not now spreading in other parts of the world?

**We do not know with any certainty whether Clade II is spreading in other parts of the world or not. Aside from North America, we are unaware of any significant surveillance efforts in other parts of the world (sampling in Europe has been rather limited to date). Thus, spread of Clade II could be occurring in the absence of documentation, especially in parts of the world where research on snake populations is uncommon. The lack of surveillance efforts (and possibility that snakes that co-evolved with this clade exhibit minimal signs of disease) also makes it difficult to know which regions Clade II occurs in naturally; thus, knowing where the fungus is native to vs introduced and spreading outside of North America cannot be determined with existing data.**

**It is also possible that Clade II has not yet spilled over into wild snake populations in other parts of the world. Keeping snakes (especially imported snakes) as pets is most common in developed countries such as the United States and several European countries. Thus, these areas are at highest risk of spillover events. Some developed countries (e.g., Australia, New Zealand, Japan) have had strict import regulations or restrict ownership of snakes as pets. This may decrease the likelihood of strains of Clade II arriving in the country in large enough numbers to cause significant spillover risk (even if the fungus has been detected in captive snakes in Australia and Japan the number of occurrences might be small enough that spillover is unlikely).**

**Because there is not enough data available on the occurrence of Clade II in wild snake populations around the world, we do not want to speculate too much in the discussion about why it has not been reported in other areas. We added the following sentence to the discussion though to highlight the importance of additional sampling to better assess where *Oo* might already have been introduced (Lines 472-475):**

**“Additional sampling would also help to better understand the global scope of *Oo* transmission between captive and wild snake populations (e.g., whether Clade II lineages have spilled over into wild snake populations in other parts of the world).”**

- What mechanisms might be offered to explain recombination, i.e. under what conditions might coinfection of different lineages within Clade II have occurred?

**This is a great question. We have added the following sentences to the discussion to address this comment (Lines 430-437):**

**“Hybridization between lineages is expected to occur more frequently in regions with high prevalence of multiple *Oo* lineages (i.e., more opportunities for co-infection with strains from distinct lineages). Relatively high prevalence (44-78%) has been reported in some snake populations [39–41], which demonstrates the potential for repeated exposure and co-infection. Factors such as host behavior, host pathogen loads, and environmental parameters, all of which likely vary based on geography, could influence transmission rates and prevalence, resulting in different frequencies of recombination among *Oo* strains.”**

Overall, this is a well-conducted and well-written study. One qualification I would like the authors and editors to note is that I do not consider myself sufficiently qualified to evaluate the specific details of the bioinformatic pipeline.

Editor: Thank you for submitting your manuscript "Population genetic analysis of Ophidiomyces ophidiicola, the causative agent of snake fungal disease, suggests recent introductions to the USA" for consideration as a Research Article at PLOS Biology. Your manuscript has been evaluated by the PLOS Biology editors, an Academic Editor with relevant expertise, and by independent reviewers. Please accept my sincere apologies for the delayed editorial process, which was due to difficulties in securing reviewers with relevant expertise.

As you will see in the reviews attached below, all reviewers appreciate the importance and accuracy of your work, and raised minor points around methodology, reporting and discussion/interpretation of the results. In light of these reviews, we are pleased to offer you the opportunity to address the comments from the reviewers in a revised version that we anticipate should not take you very long. We will then assess your revised manuscript and your response to the reviewers' comments and we may consult the reviewers and the Academic Editor again.

**We greatly appreciate this opportunity.**

We also request that your please address the following data and other policy-related requests:

1) Title - we suggest minor changes for clarity and appeal: "The population genetics of the causative agent of snake fungal disease suggests recent introductions to the USA".

**We have modified the title as suggested. We agree that this version enhances clarity.**

2) Article type change: We request that your article is converted into a *Short Report*. Short Reports present the results from a limited set of experiments that can generally be summarized in 3-4 figures or fewer. The outcomes should be self contained, rather than fitting within the narrative arc of a larger research project or article. To meet our format requirements for Short Report articles, please reduce the number of main figures to 4 maximum by either combining some of the existing ones into a single multi-panel figure, or by moving one or more to the supplementary material. Please also ensure that you select the 'Short Report' article type in the online submission form when you submit your revisions.

**As requested, we have reduced the number of main figures to four by moving one of the main figures to Supplemental Material (previous Figure 3, map).**

3) Data: you may be aware of the PLOS Data Policy, which requires that all data be made available without restriction.

Note that we do not require all raw data. Rather, we ask for all individual quantitative observations that underlie the data summarized in the figures and results of your paper

**All of the data underlying our figures has been deposited in an Open Science Framework repository (**[**https://osf.io/fmbh5/**](https://osf.io/fmbh5/)**) and we have added citations to this repository within each of the Figure legends.**
